# Clinical Efficacy of Allogeneic Bone Plate Shell Technique for Alveolar Ridge Augmentation: A Systematic Review

**DOI:** 10.7759/cureus.99228

**Published:** 2025-12-14

**Authors:** Silvia Saveinai, Akshaya Banodkar, Rajesh Gaikwad, Harshad Jain, Shushrusha Shirsat, Garima Dixit, Akhrienuo Kiso, Madhumitha Chidambaram, Nandini Metaliya

**Affiliations:** 1 Periodontology and Oral Implantology, Government Dental College and Hospital, Mumbai, IND; 2 Oral Pathology and Microbiology, Government Dental College and Hospital, Mumbai, IND

**Keywords:** allogeneic bone plate, alveolar ridge augmentation, bone regeneration, dental implants, shell technique

## Abstract

The allogeneic bone plate shell technique has emerged as a minimally invasive alternative to autogenous block grafting for alveolar ridge augmentation, aiming to reduce donor-site morbidity while maintaining predictable hard-tissue outcomes. This systematic review evaluated the clinical efficacy, dimensional stability, and implant performance of allogeneic cortical plates used with the shell technique in human patients requiring ridge augmentation before implant placement. A comprehensive electronic search across PubMed/MEDLINE, Scopus, Web of Science, Embase, EBSCOhost, and Google Scholar was conducted from inception to June 2025. Studies were eligible if they involved adult patients treated with an allogeneic shell-type augmentation and reported at least one of the following outcomes: linear or volumetric bone gain, graft resorption, implant survival, marginal bone loss, histological findings, or complications. Seven clinical studies met the inclusion criteria, comprising retrospective and prospective cohorts, case series, and one retrospective comparative study, with sample sizes ranging from 10 to 372 patients and more than 500 augmentation procedures in total. The technique was predominantly applied to horizontal or combined horizontal/vertical defects in the maxilla and mandible using thin allogeneic cortical plates fixed with microscrews and compartments filled with autogenous chips and/or xenogeneic granules. Reported horizontal gains generally ranged from approximately 2.4 to 4.8 mm, with graft resorption as low as 0.2 mm or 8%-9% in some series. Implant survival consistently ranged from 99.4% to 100%, and complications such as dehiscence or plate mobility were infrequent and usually manageable. Within the limitations of non-randomized evidence and clinical heterogeneity, allogeneic shell augmentation appears to provide predictable ridge reconstruction and high implant survival while eliminating autogenous donor-site morbidity.

## Introduction and background

Tooth loss due to trauma, periodontal disease, or extensive dental caries is closely associated with impaired oral function and reduced quality of life [1]. Dental implants have become the cornerstone of modern restorative dentistry, providing a predictable and durable option for replacing missing teeth [2]. Their success relies on predictable osseointegration between the implant surface and surrounding bone, supported by advances in implant macro- and micro-design, surfaces, and surgical protocols [3]. Most contemporary systems are fabricated from biocompatible titanium or titanium alloys, and more recently zirconia, both of which support osseointegration and functional loading [4,5]. Regardless of the specific material, long-term success depends on prosthetically driven implant placement within adequate alveolar bone volume and quality, making careful assessment of the residual ridge a critical step in treatment planning [6].

Following tooth extraction, the alveolar ridge undergoes progressive dimensional reduction, with pronounced horizontal and vertical resorption particularly within the first 6 to 12 months [7]. In many anterior maxillary and posterior atrophic sites, this loss compromises ideal three-dimensional implant positioning, so ridge augmentation is frequently required before or in conjunction with implant placement [8]. This clinical need has driven the development of multiple augmentation procedures and grafting materials to restore the ridge to prosthetically favorable dimensions [9]. Ridge augmentation is therefore a foundational component of implant site development [10], with goals that extend beyond bone volume restoration to include the creation of harmonious soft-tissue contours in esthetically demanding regions [11].

Depending on defect morphology and local anatomy, augmentation strategies may include guided bone regeneration, block grafting, distraction osteogenesis, and ridge splitting, selected according to defect type, anatomical constraints, and clinician experience [12]. Among these options, autogenous bone grafting has long been regarded as the biological “gold standard” because it provides osteogenic cells as well as osteoinductive and osteoconductive properties [10]. Classic onlay block and split-bone block concepts, such as the Khoury shell technique, harvest thin cortical plates from intraoral donor sites, fix them a short distance from the recipient bed with microscrews, and fill the created compartment with particulate autograft, achieving predictable horizontal and vertical ridge augmentation in many clinical series. However, autogenous grafts are limited by donor-site morbidity, restricted graft volume, and increased surgical complexity [13].

To overcome these drawbacks, alternative grafting options, including allogeneic, xenogeneic, and synthetic bone substitutes, have been explored [14]. Allogeneic bone grafts, derived from human donors, provide a scaffold capable of supporting new bone formation while eliminating intraoral donor-site harvesting [15]. The allogeneic shell technique, an adaptation of the Khoury method, applies the same biological concept using pre-formed or customized cortical plates from cadaveric sources to construct a rigid containment shell, which is then filled with particulate autogenous, allogeneic, or xenogeneic material to promote regeneration [13,16]. These plates offer mechanical strength and space-maintaining capacity while minimizing immunologic risk through stringent processing and sterilization [17]. Their use is particularly attractive in complex ridge defects and esthetically sensitive regions, where computer-aided design and computer-aided manufacturing (CAD/CAM) workflows can be integrated to virtually plan the augmentation and guide plate adaptation and placement [11,15].

Early clinical reports and case series on the allogeneic shell technique have described favorable horizontal bone gains, high implant survival, and, where assessed, histologic evidence of vital remodeled bone incorporating residual graft particles [18,19]. Nevertheless, concerns persist regarding the speed of revascularization, the risk of soft-tissue dehiscence or plate exposure, and the extent of long-term graft resorption [20]. In addition, most available data are derived from observational designs with heterogeneous defect types, graft combinations, and follow-up durations, which limits the strength of the conclusions that can be drawn. As interest grows in minimally invasive, donor-site-sparing, and digitally guided augmentation protocols, a focused synthesis of the clinical performance of allogeneic cortical plates used with the shell technique is warranted [21].

In this context, the present systematic review aims to evaluate the clinical efficacy of allogeneic bone plates using the shell technique for alveolar ridge augmentation. The review focuses on outcomes such as clinical bone gain, graft resorption, implant survival, marginal bone loss, and complications, with the goal of providing an evidence base to guide surgical decision-making and inform future clinical protocols.

## Review

Methodology

Protocol and Registration

The present systematic review was designed and reported in accordance with the Preferred Reporting Items for Systematic Reviews and Meta-Analyses (PRISMA) 2020 guidelines [22]. The protocol was prospectively registered in the PROSPERO international database (registration number: CRD42024614180) before the literature search and study selection commenced. The primary aim was to evaluate the clinical efficacy of allogeneic bone plates used with the shell technique for alveolar ridge augmentation. Specifically, the review sought to summarize outcomes such as clinical bone gain in horizontal and/or vertical dimensions, the extent of graft resorption, implant survival and marginal bone loss, histological findings where available, and procedure-related complications including soft-tissue dehiscence, plate exposure, or infection

Eligibility Criteria and PICOS Framework

Eligibility criteria were defined a priori using the PICOS framework to ensure a focused and clinically relevant study selection [23]. The population of interest comprised adult patients aged 18 years or older who presented with alveolar ridge deficiencies requiring augmentation prior to or in preparation for dental implant placement. The intervention was restricted to the use of the shell technique with allogeneic cortical bone plates, either alone or in combination with particulate autogenous and/or xenogeneic graft materials. The presence of a comparator was not mandatory for inclusion, but, where present, could consist of alternative augmentation approaches such as autogenous shell techniques, guided bone regeneration, or other grafting concepts. To be eligible, studies were required to report at least one of the predefined outcomes, including linear or volumetric bone gain, quantitative or qualitative graft resorption, implant survival or success, marginal bone loss, histological integration or remodeling, or clinically relevant complications.

Eligible study designs included randomized controlled trials, non-randomized controlled clinical studies, prospective or retrospective cohort studies, and case series with 10 or more patients or augmentation sites. Studies were excluded if they were in vitro or animal experiments, narrative or systematic reviews, editorials, expert opinions, technical notes, single case reports, or case series with fewer than 10 patients. Reports in which the shell concept could not be clearly attributed to allogeneic cortical plates, for example, generic onlay grafting without plate-based containment, were also excluded. Studies involving simultaneous implant placement were not included when outcomes related specifically to augmentation stability or dimensional changes could not be separated from those associated with immediate implant insertion. Only human studies published in English were considered.

Information Sources and Search Strategy

A comprehensive electronic search across PubMed/MEDLINE, Scopus, Web of Science, Embase, and EBSCOhost was conducted from inception to June 2025. No restriction was applied on publication year. Google Scholar was used as an additional source to identify potentially relevant clinical reports or case series that might not be indexed in the major databases. To further enhance completeness, the reference lists of all included studies and of relevant narrative or systematic reviews were manually screened to identify any additional eligible articles. Grey literature sources, clinical trial registries, conference proceedings, and theses were not systematically searched, and this is acknowledged as a limitation of the review.

The search strategy combined controlled vocabulary and free-text terms related to allogeneic bone, cortical plates, and shell techniques. An initial focused strategy using terms such as “allogeneic bone plate”, “cortical plate”, “shell technique”, and “alveolar ridge augmentation” was first developed and piloted in PubMed and then adapted to the syntax of the other databases [22]. As this narrow strategy resulted in a relatively small set of records, broader combinations of terms, including “allogeneic”, “cortical”, “shell”, “plate”, and “bone graft” were subsequently implemented to increase sensitivity without losing specificity. In Google Scholar, the first 300 records returned by relevance ranking were screened to identify additional clinical series using allogeneic cortical plates in a shell configuration. The full database-specific search strings, including Boolean operators and field tags, together with the exact search dates, are provided in Supplementary material 1 to facilitate reproducibility.

Study Selection

All search results were imported into reference management software, where automatic and manual procedures were used to identify and remove duplicate records. Study selection was performed in two stages. In the first stage, two reviewers independently screened the titles and abstracts of all unique records against the predefined inclusion and exclusion criteria. Citations that were clearly irrelevant to the topic or did not meet the PICOS-defined criteria were excluded at this stage. In the second stage, full-text articles were obtained for all studies deemed potentially eligible. The same two reviewers independently assessed these full texts for final inclusion. Any discrepancies between reviewers in either the title-abstract screening or full-text eligibility assessment were resolved through discussion. When consensus could not be reached, a third senior reviewer acted as arbiter.

Data Collection Process

Data extraction was conducted independently by two reviewers using a standardized and piloted Microsoft Excel form (Microsoft Corp., Redmond, WA, USA). This form was designed to capture bibliographic information (including first author, year of publication, and country), study design and setting, and key patient characteristics such as sample size, age distribution, and gender where reported. Information regarding defect characteristics (location in the maxilla or mandible; horizontal, vertical, or mixed defect morphology; and any reported defect classification) was recorded. For the intervention, details such as the type and origin of the allogeneic cortical plate, plate thickness where available, fixation method, flap design, the use of barrier membranes, and the nature of the particulate filler material were documented.

Where applicable, details of comparator interventions, such as autogenous shell grafts, guided bone regeneration (GBR), or sticky bone concepts, were also extracted. Follow-up duration, primary and secondary outcomes, and their measurement methods were recorded, including linear or volumetric assessments of bone gain, estimates of graft resorption, marginal bone loss around implants, implant survival or success rates, histological findings, and the type and frequency of complications. In cases where data were incomplete or unclear, attempts were made to contact the corresponding authors for clarification. Any disagreements between the two extractors were discussed and resolved by consensus after revisiting the original full text.

Risk-of-Bias Assessment

Risk of bias was assessed at the study level using tools appropriate to the underlying study design. For non-randomized comparative studies and cohort studies, the Risk of Bias in Non-Randomized Studies - of Interventions (ROBINS-I) tool was employed [24]. This tool evaluates seven domains of potential bias, including confounding, selection of participants, classification of interventions, deviations from intended interventions, missing data, measurement of outcomes, and selection of the reported result. The detailed review-specific questions used for assigned grades for each domain in the ROBINS-I tool are listed in Supplementary material 2. Each domain and the overall study were judged as having low, moderate, serious, or critical risk of bias, or as having insufficient information for assessment.

For case series, the Joanna Briggs Institute (JBI) Critical Appraisal Checklist for Case Series was used [25]. This checklist considers whether the inclusion criteria are clearly defined, whether participants are included consecutively or representatively, whether demographic and clinical information is adequately described, whether interventions and outcomes are clearly detailed, whether follow-up is complete and of sufficient duration, and whether the statistical analysis is appropriate. Items are scored as “Yes,” “No,” “Unclear,” or “Not applicable,” and overall risk was categorized as low, low-moderate, or moderate based on the pattern of responses. The detailed review-specific questions used for assigned grades for each domain in the case series included in the present systematic review are listed in Supplementary material 3.

All risk-of-bias assessments were performed independently by two reviewers, who then compared their judgments. Disagreements were resolved by discussion, and a third reviewer was consulted in cases where consensus was not readily achieved. In the synthesis and interpretation of the findings, greater emphasis was placed on studies judged to be at lower overall risk of bias, and this qualitative weighting is indicated where relevant in the narrative.

Synthesis Methods

Substantial clinical and methodological heterogeneity was anticipated and subsequently confirmed among the included studies, particularly in terms of defect morphology, graft compositions, surgical protocols, outcome definitions, and follow-up periods. Consequently, a quantitative meta-analysis was not undertaken. In line with the PRISMA 2020 guidelines on transparent reporting of synthesis methods, the decision to restrict the review to descriptive and narrative synthesis is explicitly stated [22].

Continuous variables such as horizontal or vertical bone gain, marginal bone loss, and linear graft resorption are reported as means with standard deviations and ranges when available. Categorical outcomes, including implant survival and the occurrence of complications, are summarized as percentages. No pooled effect estimates were calculated, and all p-values and confidence intervals reported in the Results section are those presented in the original primary studies; no re-analysis of raw data was performed.

For the narrative synthesis, studies were grouped and compared according to key features such as type of ridge defect (horizontal, vertical, or mixed), augmentation concept (allogeneic shell alone versus combinations with autogenous or xenogeneic fillers), presence or absence of a comparator technique, and the use of conventional versus CAD/CAM-guided workflows. This approach allowed structured comparison of clinical bone gain, volumetric stability, implant survival, histological integration, and complication profiles across different clinical scenarios in which the allogeneic shell technique was applied

Results

Study Selection and Characteristics

**Figure 1:**
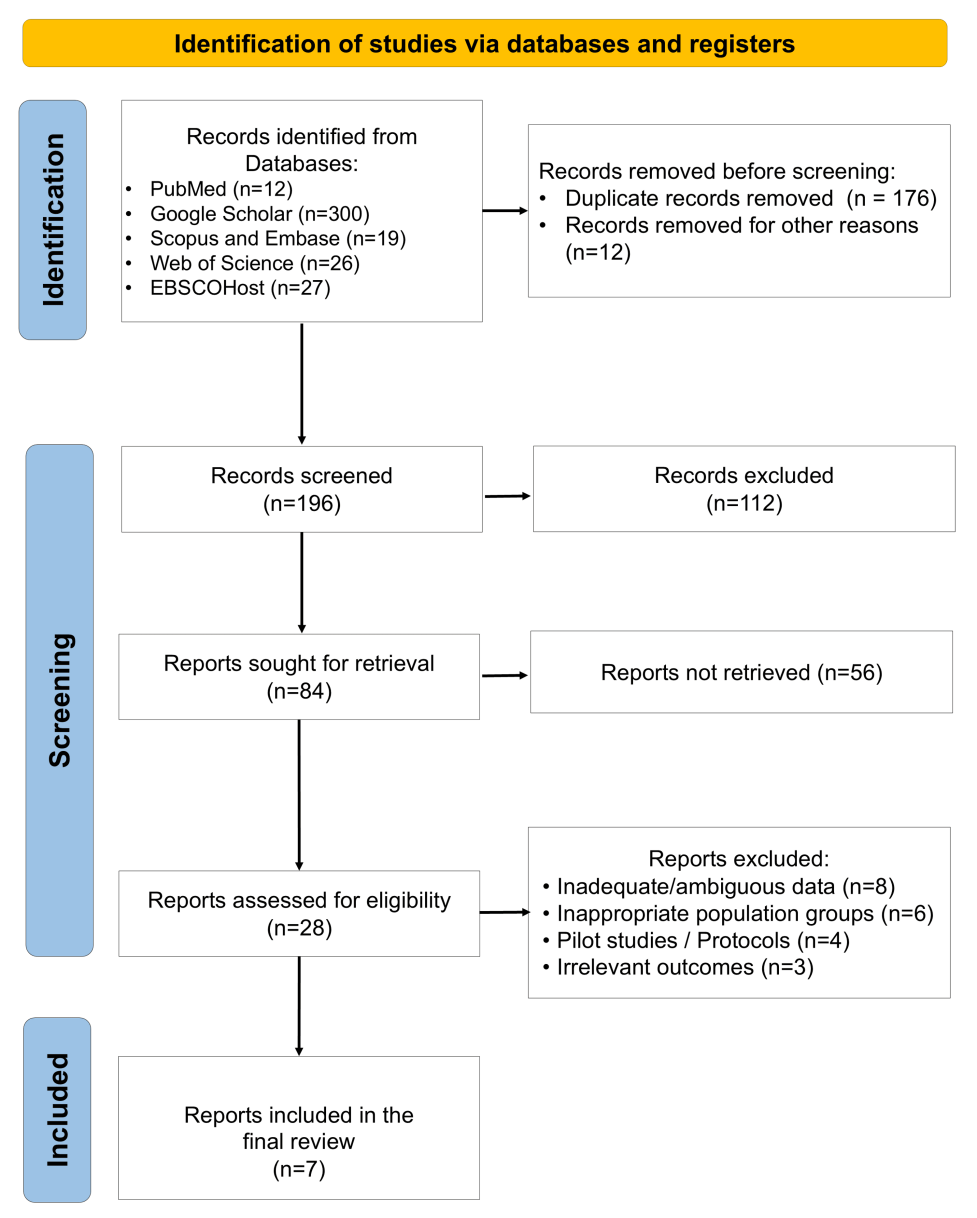
PRISMA flow diagram showing the article selection process in the present systematic review

The electronic and manual searches yielded 384 records. After removal of duplicates and screening of titles and abstracts, a smaller subset underwent full-text assessment against the predefined eligibility criteria. Seven clinical studies met the inclusion criteria and were included in this systematic review [26-32]. The study selection process is summarized in the PRISMA flow diagram (Figure 1). 

The main characteristics of the included studies are presented in Table 1. Overall, the evidence base comprised a mix of retrospective cohort designs, prospective observational or evaluative studies, and case series, as well as one retrospective comparative study [26-32]. Sample sizes ranged from 10 to 372 patients, representing both small exploratory investigations and large multicenter cohorts.

**Table 1:** Data extracted from the included studies AB: autogenous bone, ABB: anorganic bovine bone, CAD/CAM: computer-aided design/computer-aided manufacturing, CBCT: cone-beam computed tomography, CGF: concentrated growth factor, CP: cortical plate, CS: cortical shell, F: female, GBR: guided bone regeneration, HE: hematoxylin and eosin, M: male, MG: Masson-Goldner, PRF: platelet-rich fibrin, SEM: scanning electron microscopy, SB: sticky bone.

Author (year)	Country	Study design	Sample size	Age (range or mean ± SD)	Gender distribution	Defect location	Defect type (horizontal/vertical/mixed)	Augmentation site	Intervention type	Comparator	Graft material used	Fixation method	Membrane use	Gap filler material	Surgical technique description	Follow-up duration	Clinical bone gain (mm)	Marginal bone loss (mm)	Graft resorption (mm)	Implant survival rate (%)	Histological outcomes	Reported complications	Outcome measurement method	Significant results	Conclusive findings
Barbu et al. (2021) [26]	Romania	Retrospective cohort study	80 patients (127 implants)	Mean age 58.3 ± 13.4 years	40 M, 40 F (approx.; test: 12 M/28 F; control: 11 M/29 F)	Maxilla and mandible	Horizontal	Alveolar ridge (edentulous ridges)	Allogeneic bone shell technique vs sticky bone (CGF-enriched graft)	Sticky bone (CGF + bone graft)	Autogenous bone chips (BS); autogenous + anorganic bovine bone (SB)	Osteosynthesis screws	PRF or pericardium membrane depending on graft type	Autogenous bone chips (BS); sticky bone (SB)	Bone-shell technique: cortico-cancellous blocks fixed with screws; sticky bone: CGF with bone chips molded and fixed with screws	42.7 ± 16.0 months	3.7 ± 1.1 mm (BS), 3.7 ± 1.2 mm (SB)	Not directly reported	Minimal; inferred from final width gain	100%	None reported	12.5% (flap dehiscence, graft infection)	CBCT at 1 mm below alveolar crest; measured at 3 timepoints by calibrated examiner	Inverse correlation between ridge width and complications (p = 0.025); gender associated with complications (p = 0.028)	Sticky bone and bone-shell technique produced comparable outcomes for horizontal ridge augmentation; both achieved sufficient crestal width for implant placement with no implant loss
Stimmelmayr et al. (2014) [27]	Germany	Case series	17 patients, 18 sites, 30 implants	Mean 46 ± 17 years	10 females, 7 males	Maxilla and mandible	Vertical	Alveolar ridge	Modified shell technique using autogenous bone	None	Autogenous bone blocks and particulate bone from mandibular ramus	Microscrews (1.0 or 1.3 mm titanium)	Resorbable collagen membrane (Bio-Gide®)	Autogenous particulate bone chips	Thin cortical bone shells placed horizontally, fixed with screws; space filled with autogenous bone chips and covered with collagen membrane	Mean 15 ± 11 months after loading	Mean vertical defect 4.8 ± 1.4 mm; resorption during consolidation 0.5 ± 0.7 mm	Not reported	Mean 0.5 ± 0.7 mm	100%	Not reported	2 cases of wound dehiscence; no implant failures	Radiographs and CBCT; measurements with radiographic software perpendicular to bone crest	Mean gain 4.8 mm; vertical resorption minimal (0.5 mm); all implants placed as planned	Modified shell technique provided predictable vertical ridge augmentation with moderate resorption, minimal complications, and 100% implant survival
Karkar et al. (2023) [28]	Egypt	Prospective evaluative study	15 patients (15 implant sites)	Mean 26.8 ± 6.09 years	9 females, 6 males	Anterior maxilla	Horizontal	Anterior aesthetic zone	Allogenic bone shell + autogenous bone chips (Khoury shell technique)	None	Allograft shell (Maxgraft® Cortico) + autogenous chips	Microscrews (1.2 mm titanium)	None reported	Autogenous bone chips	Allograft shell fixed labially with microscrews; gap filled with autogenous chips	6 months	Apical: 2.64 ± 0.99 mm; mid-level: 3.44 ± 0.52 mm; crestal: 2.36 ± 0.85 mm	Not directly reported	Estimated 1-1.5 mm crestally (not directly measured)	Not numerically reported; all implants successfully placed except 1 case	Vital bone with osteocytes, osteoblasts, reversal and resting lines	1 infection with graft loss, 3 shell detachments	CBCT at apical, mid, and crestal points	Statistically significant bone gain (p < 0.0001 at mid-level)	Khoury shell technique using allogenic shell and autogenous chips showed reliable augmentation with low complication rates
Cinar et al. (2022) [29]	Turkey	Retrospective comparative study	36 patients (18 CS group, 18 GBR group)	Mean 51.7 years (range 38-65)	17 females, 19 males	Maxilla (anterior and posterior)	Horizontal	Horizontally deficient alveolar ridges	Allogeneic CS shell technique + 1:1 autogenous + ABB	GBR with same graft mix but without CS	1:1 Autogenous bone + Anorganic bovine bone (ABB)	CS: 2 osteosynthesis screws; GBR: no fixation	Resorbable collagen membrane (Jason®)	Autogenous + ABB (1:1)	Decortication, graft packed into defect; CS fixed with screws in test group	7 months	Volumetric gain: CS: 5790.80 → 5344.49 mm³; GBR: 5741.23 → 4872.38 mm³	Not reported	CS: 8.26 ± 1.60%, GBR: 14.36 ± 3.55%	100%	Not evaluated	None in either group	CBCT volumetric analysis using MIMICS	CS group had significantly lower bone resorption (p < 0.001)	CS shell technique showed stable graft volume and similar implant success as GBR
Tunkel et al. (2023) [30]	Germany	Prospective observational clinical trial	117 patients, 229 implants	Mean 56 ± 12.7 years (range 19-84)	83 females, 34 males	Maxilla and mandible	Mixed (Class II-IV defects)	Segmental jaw atrophy sites	Allogeneic vs autogenous shell technique	Autogenous shell, mixed (both allogeneic and autogenous)	Autogenous bone chips and/or allogeneic cortico-cancellous granules	At least 2 adjusting screws (1 mm microscrews)	Collagen membrane (Jason® membrane)	Autogenous bone or mixture with allogeneic granules	Shells shaped, fixed, filled with graft, closed with flap, implants after 4-6 months	Mean 9 months	Not quantified directly; surgical efficiency and implant placement outcomes reported	Not reported	Not reported	99.6%	Not assessed	Intraoperative: 10.3%; postoperative: 8.5%	Surgery time recorded; complications and survival monitored	Shorter surgery time with allogeneic shell (p < 0.001); more complications with combined use and sinus lift (p = 0.02)	Allogeneic shell technique offers shorter surgery time and similar complication rates, supporting clinical use
Kämmerer et al. (2022) [31]	Germany	Multicenter case series	372 augmentation cases; 656 implants	Not reported	Not reported	Maxilla and mandible	Horizontal and horizontal/vertical	Alveolar ridge defects (Class II-IV)	Allogeneic cortical plate (CP) with shell technique	None	Autogenous, allogeneic, mixture with xenogeneic granules	Adjusting screws (1.0-1.5 mm diameter)	Porcine collagen membrane (e.g., Jason® or Bio-Gide®)	Autogenous/allogeneic/bovine xenograft granules	CP trimmed, fixed, filled with granules, membrane placed; implant after 4-6 months	1-12 years (mean 3.5 years)	Not reported numerically; all successful cases adequate for implants	Not reported	Not reported; histological remodeling observed	99.4%	Advanced osteogenesis, embedded allograft remnants; no inflammation	8.1% (dehiscence, plate fracture, screw exposure); implants still placed in most	Clinical, CBCT, histology (HE/MG staining), SEM	High implant success despite complications; full graft remodeling observed	Allogeneic shell technique effective for ridge augmentation with good outcomes and minimal morbidity
Doliveux and Doliveux al. (2024) [32]	France and Canada	Prospective case series	10 patients, 12 grafted sites	Not reported	Not reported	Anterior maxilla	Horizontal	Alveolar ridge (anterior esthetic zone)	Allogeneic cortical plate (Maxgraft Cortico) + autogenous bone chips using CAD/CAM-guided shell technique	None	Allogeneic cortical plate + autogenous bone chips	1-mm stainless steel microscrews (mean 2 ± 0.2 per site)	Not used	Autogenous bone chips	Digitally planned shell plates and surgical guides; plates fixed and gap filled with autogenous bone	Up to 2 years	Crest width: 2.4 ± 1.0 mm → 7.9 ± 0.6 mm (postop), 7.6 ± 0.6 mm (4 months)	0.07 ± 0.13 mm (1 yr), 0.13 ± 0.17 mm (2 yrs)	0.2 ± 0.1 mm at 4 months	100%	Macroscopic revascularization observed; no direct histology	3 plates had slight mobility; no dehiscence or perforation	CBCT and periapical radiographs	High volume stability (0.2 mm resorption), minimal marginal bone loss	CAD/CAM-guided allogeneic shell technique simplified surgery, lowered morbidity, and provided predictable graft and implant stability

Geographically, three studies were conducted in Germany, one in Romania, one in Egypt, one in Turkey, and one was a binational series from France and Canada [26-32]. Mean patient age, where reported, ranged from approximately 26.8 years in a young anterior maxillary cohort to over 58 years in broader mixed dentitions, and both male and female patients were represented across studies, although detailed gender distribution was not uniformly specified [26-32].

Most studies focused on alveolar ridge augmentation to facilitate subsequent implant placement. Five investigations primarily addressed horizontal ridge deficiencies in the maxilla and/or mandible, often with particular emphasis on anterior esthetic zones [26,28-32]. One study concentrated on vertical defects treated with a modified shell technique using autogenous bone [27], and another addressed horizontally deficient ridges using either an allogeneic cortical shell or GBR with the same particulate graft composition [29]. Defect classes ranged from localized post-extraction deficiencies to segmental jaw atrophy (Class II-IV defects) [30,31].

Surgical Technique and Materials

All included studies employed a shell-type augmentation concept using thin cortical plates shaped and fixed to the recipient site to create a rigid compartment, which was then filled with particulate graft material. In the key allogeneic protocols, the shell consisted of processed cortical plates from human donor bone, secured with 1.0-1.5 mm titanium osteosynthesis or microscrews [26,28-32]. The gap between the plate and the residual ridge was most commonly filled with autogenous bone chips harvested intraorally, with several studies also using combinations of autogenous bone and xenogeneic granules, particularly anorganic bovine bone (ABB) in a 1:1 ratio [26,29,31].

One comparative study evaluated allogeneic cortical shells against GBR using an identical particulate mixture of autogenous bone and ABB but without a cortical plate in the control group [29]. Another prospective trial compared allogeneic shells with autogenous shells and mixed combinations in segmental jaw atrophy, focusing on operative time and complication rates [30]. Collagen membranes (e.g., Jason® or Bio-Gide®) were used in several studies to cover the grafted region, although at least one report described favorable outcomes without additional membrane coverage [26,29,31]. In a digitally guided protocol, Doliveux and Doliveux [32] employed CAD/CAM-designed allogeneic plates and surgical guides to pre-shape the shell, aiming to reduce intraoperative adjustments and improve the accuracy of the final ridge contour.

Bone Gain and Ridge Dimensional Changes

Measures of bone gain were reported in linear or volumetric terms, with horizontal augmentation being the most frequently documented parameter. Barbu et al. [26] reported mean horizontal width gains of approximately 3.7 ± 1.1 mm in the bone-shell group and 3.7 ± 1.2 mm in the sticky-bone group, providing sufficient crestal width for implant placement. In the anterior maxilla, Karkar et al. [28] documented mean horizontal increases of 2.64 ± 0.99 mm apically, 3.44 ± 0.52 mm at mid-root level, and 2.36 ± 0.85 mm crestally at six months, with statistically significant improvement at the mid-root level (p < 0.0001 as reported by the authors).

Cinar et al. [29] used CBCT-based volumetric analysis and showed that both the cortical shell and GBR groups achieved substantial ridge volume increases; however, the shell group demonstrated better volume preservation over seven months, with mean resorption of 8.26 ± 1.60% compared with 14.36 ± 3.55% in the GBR group (p < 0.001, as reported by the authors). Doliveux and Doliveux [32] observed substantial crestal width augmentation in the anterior maxilla, from 2.4 ± 1.0 mm preoperatively to 7.9 ± 0.6 mm immediately postoperatively, with only slight reduction to 7.6 ± 0.6 mm after four months when using CAD/CAM-guided allogeneic shells.

Although primarily focused on autogenous bone, the vertical augmentation achieved in the modified shell technique reported by Stimmelmayr et al. [27] provides a useful reference: a mean vertical defect of 4.8 ± 1.4 mm was corrected with a mean resorption of 0.5 ± 0.7 mm during consolidation, illustrating the magnitude of vertical reconstruction achievable with a shell approach. In the multicenter series by Kämmerer et al. [31], detailed linear measurements were not uniformly reported, but clinical descriptions indicated that sufficient ridge width and height were obtained in nearly all cases to allow planned implant placement.

Graft Resorption and Marginal Bone Loss

Graft resorption was described either as a linear change in width or height or as a percentage of initial graft volume. The lowest reported linear resorption was 0.2 ± 0.1 mm at four months in the CAD/CAM-guided series by Doliveux and Doliveux [32], indicating excellent early volume stability. In the comparative analysis by Cinar et al. [29], volumetric resorption was significantly lower in the cortical shell group than in the GBR group, reinforcing the space-maintaining effect of a rigid cortical plate.

Marginal bone loss around implants was reported in a limited number of studies but remained minimal where documented. Doliveux and Doliveux [32] observed marginal bone loss of 0.07 ± 0.13 mm at one year and 0.13 ± 0.17 mm at two years, suggesting stable peri-implant bone levels following augmentation with allogeneic shells in the esthetic zone. In other reports, marginal bone levels were described qualitatively as stable, without clinically relevant loss jeopardizing implant prognosis [26,31].

Implant Survival

Implant survival rates were uniformly high across the included studies. Barbu et al. [26] reported 100% implant survival in both the allogeneic bone-shell and sticky-bone groups during a mean follow-up of 42.7 ± 16.0 months. In the vertical autogenous shell series by Stimmelmayr et al. [27], all 30 implants survived over a mean follow-up of 15 ± 11 months after loading.

In the comparative study by Cinar et al. [29], implant survival reached 100% in both the cortical shell and GBR groups. The large multicenter cohort presented by Kämmerer et al. [31] documented a survival rate of 99.4% for 656 implants placed following allogeneic shell augmentation over a follow-up period of 1-12 years (mean 3.5 years). Similarly, Tunkel et al. [30] reported 99.6% implant survival in 229 implants placed after either allogeneic or autogenous shell augmentation. Doliveux et al. [32] also recorded 100% implant survival in 12 grafted sites up to two years. Collectively, these data suggest that the allogeneic shell technique does not compromise implant survival and is comparable to autogenous shell grafting in this respect [26-32].

Complications

Reported complication rates were generally low and primarily related to soft-tissue healing and plate stability. In the Barbu et al. cohort [26], complications such as flap dehiscence and graft infection occurred in approximately 12.5% of cases, but these events did not lead to implant loss. Karkar et al. [28] reported one infection with graft loss and three cases of shell detachment, all managed without long-term compromise of implant placement.

Kämmerer et al. [31] observed an overall complication rate of 8.1%, including dehiscence, plate fracture, and screw exposure; despite these issues, implants could still be placed in the majority of affected sites. In the prospective trial by Tunkel et al. [30], intraoperative complications occurred in 10.3% of cases and postoperative events in 8.5%, with more frequent complications associated with combined procedures, such as simultaneous sinus lift, rather than with the allogeneic shell material per se. None of the included studies reported catastrophic failures or complications that precluded successful implant rehabilitation when appropriate management was undertaken [26-32].

Histological and Radiologic Findings

Histological and radiologic assessments, where available, supported the clinical impression of favorable integration of allogeneic shells. Karkar et al. [28] reported vital bone containing osteocytes and osteoblasts, with reversal and resting lines indicative of active remodeling. Kämmerer et al. [31] described advanced osteogenesis with embedded allograft remnants and an absence of inflammatory cell infiltrates on histology and scanning electron microscopy, suggesting good biocompatibility and incorporation of the allogeneic plates.

Radiographically, cone-beam computed tomography (CBCT) and periapical radiographs were used to assess ridge dimensions and peri-implant bone levels. Doliveux and Doliveux [32] documented macroscopic revascularization of the grafted region and high volume stability in their CAD/CAM-guided cases. Barbu et al. [26] and Cinar et al. [29] used CBCT-based linear and volumetric measurements to quantify bone gain and resorption, demonstrating stable ridge contours at follow-up.

Summary of Key Findings

In summary, seven clinical studies from six countries evaluated the use of allogeneic cortical plates in a shell configuration for alveolar ridge augmentation, encompassing 10-372 patients per study [26-32]. Most investigations addressed horizontal ridge deficiencies, with mean linear bone gains typically between 2.36 mm and 4.8 mm and very limited resorption, sometimes as low as 0.2 mm in early follow-up [26-29,32]. Where volumetric analysis was performed, allogeneic shells showed significantly lower resorption than GBR while maintaining comparable implant success [29].

Implant survival consistently ranged from 99.4% to 100% across the dataset, and reported complications were relatively infrequent and manageable, rarely jeopardizing implant placement or function [26-32]. Histological and radiologic observations supported the notion of progressive graft remodeling and stable peri-implant bone levels, particularly in the context of CAD/CAM-guided shells [28,31,32]. Collectively, these findings indicate that allogeneic bone plates used with the shell technique provide predictable ridge augmentation with high implant survival, good volumetric stability, and an acceptable complication profile, and appear comparable to autogenous shell techniques while avoiding donor-site morbidity.

Risk-of-Bias Assessment

Risk-of-bias assessments for the case series, performed using the JBI Critical Appraisal Checklist, are summarized in Table 2. All three case series [27,31,32] clearly reported clinical objectives, patient characteristics, interventions, and outcomes. The main limitation related to potential non-consecutive or unclear sampling in the large multicenter series by Kämmerer et al. [31], resulting in an overall judgment of low to low-moderate risk of bias for the case series evidence base.

**Table 2:** Risk-of-bias assessment in case series using the JBI Critical Appraisal Tool

Study	Q1: Criteria clearly defined	Q2: Condition measured reliably	Q3: Consecutive inclusion	Q4: Complete inclusion	Q5: Demographics reported	Q6: Clinical information	Q7: Follow-up complete	Q8: Outcome reporting	Q9: Statistical analysis	Overall Risk
Stimmelmayr et al. (2014) [27]	Yes	Yes	No	Yes	Yes	Yes	Yes	Yes	Yes	Low
Kämmerer et al. (2022) [31]	Yes	Yes	Unclear	Yes	Yes	Yes	Yes	Yes	Yes	Low to moderate
Doliveux and Doliveux (2024) [32]	Yes	Yes	Yes	Yes	Yes	Yes	Yes	Yes	Yes	Low

**Figure 2:**
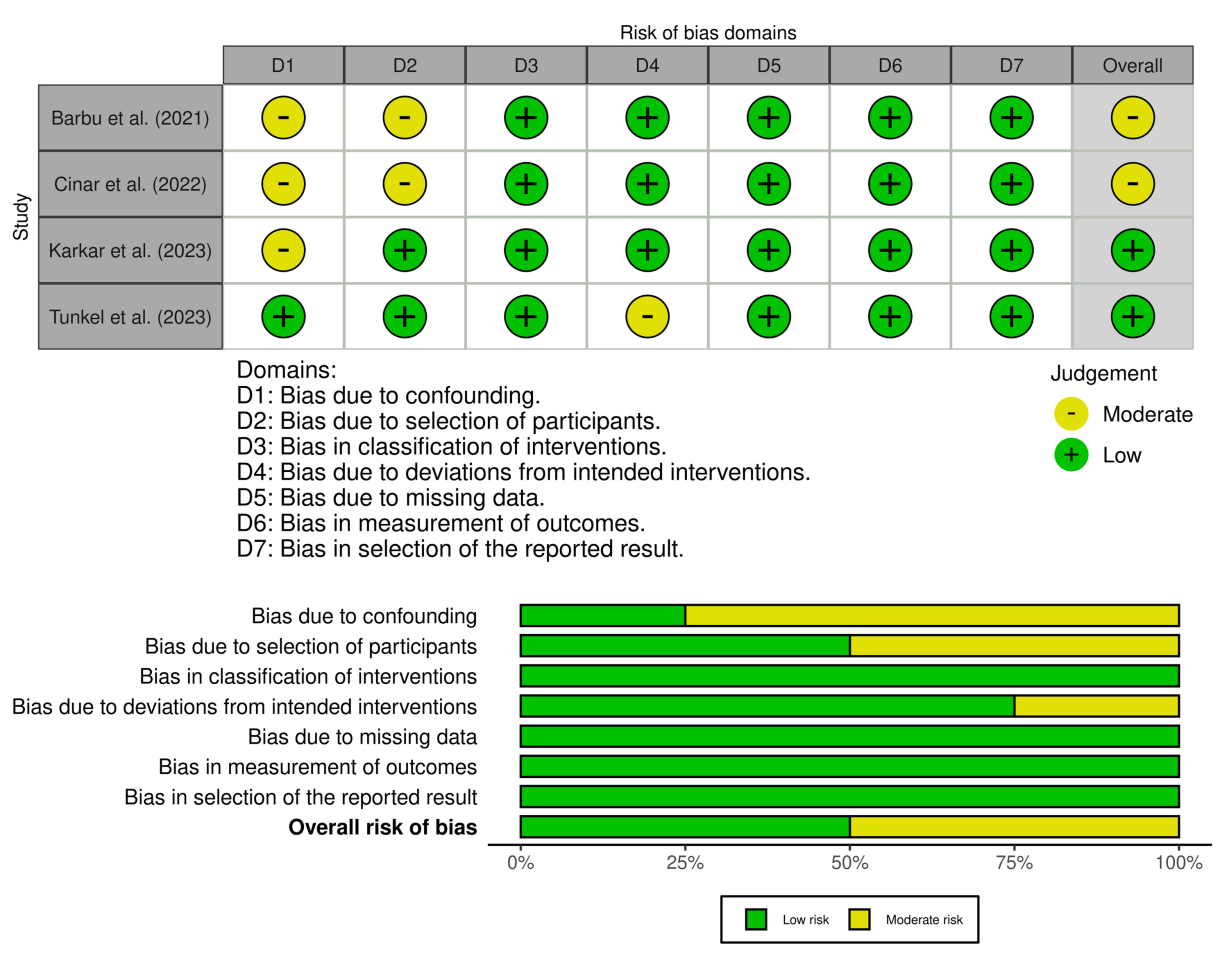
Risk of bias across non-randomized clinical trials using the ROBINS-I tool Source: [26,28-30].

For the non-randomized prospective and retrospective comparative studies, risk of bias was evaluated using the ROBINS-I tool [24]. Most studies were judged to have a moderate overall risk of bias, driven chiefly by the lack of randomization, potential confounding, and variability in clinical protocols [26,28-30]. Nevertheless, outcome measurement methods were generally appropriate and clearly described, and follow-up periods were adequate to capture early and medium-term augmentation outcomes. Among these, the prospective trial by Tunkel et al. [30] exhibited the lowest overall risk, with only mild concern about deviations from intended interventions related to differences in execution across participating sites. The distribution of risk-of-bias judgments across domains is illustrated in Figure 2.

Discussion

Summary of Principal Findings

This systematic review synthesized data from seven clinical studies that evaluated the use of allogeneic cortical bone plates in a shell configuration for alveolar ridge augmentation [26-32]. Across these heterogeneous but consistently positive reports, the technique was used predominantly for horizontal or combined horizontal/vertical defects in both maxilla and mandible, with a particular emphasis on anterior esthetic zones [26-32]. Horizontal bone gain typically ranged between approximately 2.36 mm and 4.8 mm, with volumetric analyses confirming stable graft volumes in the medium term [26-29,32]. Reported graft resorption was generally low, in some cases as little as 0.2 mm or 8%-9% of the initial graft volume [29,32]. Implant survival was uniformly high, between 99.4% and 100%, even in cohorts that included complex Class II-IV defects [27,30,31]. Complications such as dehiscence, plate mobility, or local infection occurred in a minority of cases and rarely jeopardized the feasibility of implant placement [26-32]. Collectively, the available evidence suggests that the allogeneic shell technique is a predictable method for ridge augmentation with favorable hard-tissue stability and implant outcomes.

Comparison With Autogenous Block Grafts and Other Augmentation Techniques

Autogenous bone remains the traditional gold standard for ridge augmentation because of its combined osteogenic, osteoinductive, and osteoconductive properties [10]. Classical onlay block grafts from intraoral donor sites and more recently refined autogenous shell techniques (Khoury concept) reliably produce clinically meaningful bone gains but at the cost of donor-site morbidity, increased operative time, and limited graft availability [10,13]. Systematic reviews of lateral ridge augmentation have reported mean horizontal gains in the order of 3.5-4.0 mm when all techniques, most commonly autogenous blocks and xenograft plus membrane, are pooled, with high implant survival but non-negligible complication and resorption rates. Sánchez-Sánchez et al. found a mean horizontal gain of about 3.9 mm for staged lateral augmentation procedures with survival rates >95%, with autogenous blocks being the most frequently used intervention [33]. Monje et al. reported mean horizontal gains close to 5 mm in severely resorbed anterior maxillae treated with autogenous symphyseal blocks and particulate xenograft but also documented clinically relevant graft resorption and donor-site morbidity [34].

The magnitude of horizontal bone gain observed in the present allogeneic shell cohort (approximately 2.36-4.8 mm) [26-29,32] therefore falls squarely within the range typically reported for autogenous blocks and lateral augmentation procedures in general. Importantly, the percentage or linear resorption described for the allogeneic shells (as low as 0.2 mm or 8.26% in some series) [29,32] appears at least comparable, and in some contexts lower, than the 15%-30% resorption frequently reported for autogenous onlay blocks over time [35]. This is clinically relevant because excessive remodeling of autogenous blocks in thin crests may compromise both implant positioning and peri-implant soft-tissue support.

When specifically compared with autogenous shell techniques, the allogeneic plate approach seems to offer similar hard-tissue performance while avoiding donor-site surgery. Autogenous Khoury-style shells have shown horizontal gains in the order of 3-5 mm with favorable stability but require harvesting thin cortico-cancellous plates from the mandibular ramus or symphysis [33]. In our included material, Tunkel et al. demonstrated that allogeneic shells achieved implant survival rates comparable to autogenous shells but with significantly shorter surgery times [30]. Karkar et al. and Doliveux and Doliveux further showed that allogeneic shells, with or without CAD/CAM customization, can generate substantial crestal width increases in the anterior maxilla with limited resorption and a low incidence of complications [28,32].

The small split-mouth case series by Tunkel et al., which directly compared autogenous and allogeneic shells in five patients, also reported comparable vertical and horizontal bone gains and similar complication profiles, supporting the notion of clinical equivalence between the two shell materials for selected defects [36]. This study did not meet our pre-specified inclusion criterion of a minimum of 10 participants per group and was therefore excluded from the main synthesis; nonetheless, its findings are consistent with the larger prospective trial by the same group [30] and help contextualize the present results.

Beyond the shell concept, a wide spectrum of regenerative approaches exists, including particulate-guided bone regeneration with xenografts and resorbable membranes, titanium meshes, distraction osteogenesis, and combined inlay-onlay grafting. Large systematic reviews and narrative overviews consistently indicate that lateral augmentation with particulate graft plus membrane and autogenous or allogeneic blocks can achieve horizontal gains of around 3-4 mm with high implant survival. Vertical augmentation is more technique-sensitive and associated with higher complication rates, particularly when using onlay blocks or titanium meshes, whereas distraction osteogenesis offers substantial vertical gains but at the price of longer treatment times and greater technical complexity [35]. Within this broader landscape, the allogeneic shell technique appears particularly well suited to moderate horizontal or combined defects where a contained space can be created, donor-site surgery is undesirable, and soft-tissue management can support tension-free closure.

Methodological Considerations and Limitations

The overall certainty of the evidence is constrained by the predominance of observational designs. Among the seven included studies, there were retrospective and prospective cohorts, case series, and one retrospective comparative study, with no randomized controlled trials [26-32]. Non-randomized designs are inherently vulnerable to selection bias, confounding, and performance bias, particularly when treatment allocation is influenced by defect severity, clinician preference, or patient-related factors. This is reflected in the ROBINS-I assessments, which generally classified the non-randomized studies as having moderate risk of bias, mainly due to potential confounding and lack of randomization [24,26,28-30]. Case series appraised with the JBI checklist were broadly judged to have low to low-moderate risk of bias, but the absence of control groups limits causal inference [25,27,31,32].

Substantial clinical and methodological heterogeneity further limits the strength of pooled conclusions. The included studies differed in defect location (anterior vs posterior, maxilla vs mandible), defect morphology (purely horizontal vs mixed or vertical), graft composition (autogenous chips alone vs mixtures with xenograft), membrane use, plate thickness, and whether digital planning/CAD-CAM customization was employed [26-32]. Follow-up periods ranged from four months to more than a decade, and outcome measures were variably reported using linear, volumetric, and radiographic indices. Horizontal bone gain was measured at different reference levels (crestal, mid-level, or apical) or indirectly inferred from final ridge width in some series. Only a subset of studies quantified marginal bone loss or graft resorption explicitly, and histological evaluation was limited to two reports [28,31,32]. In view of this heterogeneity and the small number of comparable data sets, a formal meta-analysis was neither feasible nor appropriate, and a descriptive synthesis was chosen, as stated in the Methods.

Publication bias and selective reporting may also have influenced the apparent success of the allogeneic shell technique. Small case series with unfavorable outcomes may be under-represented in the literature, and complications could be under-reported or categorized inconsistently. Furthermore, most data are derived from specialized centers with advanced surgical expertise; the extent to which similar results can be replicated in routine practice or by less experienced operators remains uncertain. Finally, while follow-up periods of up to 12 years were reported in one large multicenter series [31], long-term data remain sparse overall, particularly with respect to peri-implant bone stability and prosthetic complications beyond the early loading period.

Clinical Implications and Case Selection

Despite these limitations, several practical implications emerge from the current synthesis. First, the magnitude of horizontal bone gain achievable with allogeneic shells is comparable to that reported for autogenous blocks and particulate GBR, but with the important advantages of avoiding donor-site morbidity and reducing operative time [26-32]. This can be particularly attractive in patients with limited intraoral donor bone, high esthetic demands, or medical conditions that make additional surgical sites undesirable. Second, the technique appears especially useful for segmental horizontal defects in the anterior maxilla and posterior regions where 3-5 mm of additional width is required for prosthetically driven implant placement [26,28,32].

The low but non-negligible rates of flap dehiscence and plate exposure underscore the importance of meticulous soft-tissue management. Studies consistently emphasize the need for tension-free primary closure, adequate flap thickness, and careful management of thin biotypes to minimize exposure risk [26-32]. In the event of minor dehiscence, several authors reported that grafts often remained stable and implants could still be placed as planned, suggesting a degree of biological and mechanical resilience [26,30,31]. Nonetheless, patient selection should remain cautious in heavy smokers, individuals with poor oral hygiene, or those with compromised soft tissues, in whom the risk of exposure and infection may be higher.

From a broader treatment-planning perspective, the allogeneic shell technique should be viewed as a complement rather than a wholesale replacement for autogenous grafts and other vertical augmentation modalities. Current evidence supports its use primarily for horizontal and combined defects of moderate severity; for extensive vertical deficiencies, sinus floor elevation, distraction osteogenesis, or autogenous onlay/inlay grafts still have a more established evidence base. Emerging CAD/CAM workflows, as illustrated by Doliveux and Doliveux [32], may further refine case selection by enabling precise three-dimensional planning, pre-shaping of plates, and guided surgery, thereby reducing intraoperative adjustment and improving predictability.

Future Research Directions

Future studies should prioritize well-designed prospective trials directly comparing allogeneic shell techniques with autogenous shells, traditional autogenous blocks, and particulate GBR in clearly defined defect categories. At a minimum, multicenter prospective cohorts with standardized inclusion criteria, defect classifications, and outcome measures are needed to reduce heterogeneity and facilitate meaningful comparisons. Randomized controlled trials with sufficient sample sizes would allow more robust inferences regarding comparative effectiveness, complication rates, and long-term implant performance. Contemporary reviews of ridge augmentation emphasize the importance of defect-specific decision-making and highlight the current gaps in high-quality comparative data between autografts, allografts, and xenografts.

Standardization of radiographic protocols (e.g., CBCT-based linear and volumetric measurements at fixed landmarks), reporting of bone gain and resorption, and inclusion of peri-implant marginal bone level changes at defined time points would substantially improve the interpretability of future work. Longer follow-up, ideally exceeding five years after loading, is needed to confirm the stability of regenerated bone and implants over time. Additionally, histologic and histomorphometric studies in humans, building on the limited evidence currently available [28,31], would further clarify the remodeling dynamics and degree of integration of allogeneic plates. Finally, patient-reported outcomes, cost-effectiveness analyses, and evaluations of learning curves and operator variability should be incorporated into future research agendas to fully characterize the role of the allogeneic shell technique within contemporary implant therapy.

## Conclusions

Within the limitations of this systematic review, it can be concluded that the allogeneic bone plate shell technique is a promising, clinically effective approach for alveolar ridge augmentation, particularly for horizontal defects in esthetically sensitive regions. The technique demonstrated consistent bone gain, minimal graft resorption, high implant survival rates, and manageable complication profiles across diverse clinical settings. By eliminating the need for autogenous donor sites, it reduces patient morbidity while maintaining structural integrity and regenerative capacity. However, the current body of evidence is primarily based on observational studies with methodological heterogeneity, underscoring the need for future high-quality randomized controlled trials with standardized outcome measures and long-term follow-up to establish its efficacy and integration into routine clinical practice fully.

## Appendices

Supplementary material 1 

**Table 3:** Search strings across various databases

Database	Search string used
PubMed (MEDLINE)	("Alveolar Ridge Augmentation"[Mesh] OR "alveolar ridge"[tiab] OR "ridge augmentation"[tiab] OR "jaw atrophy"[tiab] OR "segmental jaw"[tiab]) AND (allogeneic[tiab] OR allograft*[tiab] OR "allogeneic bone"[tiab] OR "allogeneic graft*"[tiab]) AND ("cortical plate"[tiab] OR "cortical plates"[tiab] OR "bone plate"[tiab] OR "bone plates"[tiab] OR "cortical shell"[tiab] OR "shell technique"[tiab] OR "shell concept"[tiab]) AND (implant*[tiab] OR "dental implant*"[tiab] OR "implant therapy"[tiab]) NOT (animals[mh] NOT humans[mh])
Embase (Ovid)	('alveolar ridge augmentation'/exp OR 'alveolar ridge':ab,ti OR 'ridge augmentation':ab,ti OR 'jaw atrophy':ab,ti OR 'segmental jaw':ab,ti) AND (allogeneic:ab,ti OR allograft*:ab,ti OR 'allogeneic bone':ab,ti OR 'allogeneic graft*':ab,ti) AND ('cortical plate':ab,ti OR 'cortical plates':ab,ti OR 'bone plate':ab,ti OR 'bone plates':ab,ti OR 'cortical shell':ab,ti OR 'shell technique':ab,ti OR 'shell concept':ab,ti) AND (implant*:ab,ti OR 'dental implant*':ab,ti OR 'implant therapy':ab,ti) AND [humans]/lim AND [english]/lim
Scopus	TITLE-ABS-KEY(("alveolar ridge" OR "ridge augmentation" OR "jaw atrophy" OR "segmental jaw") AND (allogeneic OR allograft* OR "allogeneic bone" OR "allogeneic graft*") AND ("cortical plate" OR "cortical plates" OR "bone plate" OR "bone plates" OR "cortical shell" OR "shell technique" OR "shell concept") AND (implant* OR "dental implant*" OR "implant therapy")) AND (LIMIT-TO(LANGUAGE, "English")) AND (LIMIT-TO(DOCTYPE, "ar"))
Web of Science (Core Collection)	TS=(("alveolar ridge" OR "ridge augmentation" OR "jaw atrophy" OR "segmental jaw") AND (allogeneic OR allograft* OR "allogeneic bone" OR "allogeneic graft*") AND ("cortical plate" OR "cortical plates" OR "bone plate" OR "bone plates" OR "cortical shell" OR "shell technique" OR "shell concept") AND (implant* OR "dental implant*" OR "implant therapy")) Refined by: DOCUMENT TYPES = (ARTICLE) AND LANGUAGES = (ENGLISH)
EBSCOhost (e.g., Dentistry & Oral Sciences Source/CINAHL)	TX(("alveolar ridge" OR "ridge augmentation" OR "jaw atrophy" OR "segmental jaw") AND (allogeneic OR allograft* OR "allogeneic bone" OR "allogeneic graft*") AND ("cortical plate" OR "cortical plates" OR "bone plate" OR "bone plates" OR "cortical shell" OR "shell technique" OR "shell concept") AND (implant* OR "dental implant*" OR "implant therapy")) AND (PT article) AND (LA English)
Google Scholar	"alveolar ridge augmentation" AND (allogeneic OR allograft) AND ("cortical plate" OR "cortical shell" OR "shell technique") AND ("dental implant" OR implant). The first 300 results sorted by relevance were screened

Supplementary material 2 

**Table 4:** ROBINS-I domains and example signaling questions used for grading the risk of bias in the present systematic review

Domain	Example signaling questions for non-randomized clinical studies
Bias due to confounding	Were important prognostic factors or baseline characteristics (e.g., defect morphology, defect location, smoking status, systemic conditions) measured and accounted for in the analysis or in the design?
	Were there any imbalances in baseline characteristics between intervention groups that could influence the outcomes (e.g., more severe defects in one group)?
Bias in selection of participants into the study	Were participants included in the study in a way that was unrelated to both intervention and outcome (e.g., consecutive or clearly defined inclusion)?
	Were eligibility criteria clearly reported and applied consistently across groups?
Bias in classification of interventions	Were the allogeneic shell, autogenous shell, GBR, or other augmentation procedures clearly defined and reliably classified?
	Is it likely that any participants were misclassified regarding the intervention received (e.g., shell vs non-shell technique)?
Bias due to deviations from intended interventions	Were augmentation procedures performed according to the pre-specified protocol (e.g., plate type, graft mix, healing time)?
	Were co-interventions (e.g., simultaneous sinus lift, membrane type, flap design) similar across groups, or were deviations likely to affect outcomes?
Bias due to missing data	Were follow-up rates adequately reported for both augmentation and implant outcomes?
	Is the amount, nature, or handling of missing data (loss to follow-up, incomplete radiographic data) likely to bias the results?
Bias in measurement of outcomes	Were outcome assessors (clinical and radiographic) blinded to the intervention group where feasible?
	Were measurement methods for bone gain, resorption, marginal bone loss, and complications standardized and validated (e.g., calibrated CBCT measurements, predefined complication criteria)?
Bias in selection of the reported result	Were all prespecified outcomes (e.g., bone gain, resorption, implant survival, complications) reported, or is there evidence of selective reporting?
	Were statistical analyses clearly described, and were all analyses reported consistently with the Methods?

Supplementary material 3 

**Table 5:** JBI Critical Appraisal Checklist for Case Series - items and interpretation

Item no.	Checklist item (case series)	Example interpretation in the present review
1	Were there clear criteria for inclusion in the case series?	Inclusion criteria for augmentation cases were predefined (e.g., ridge defects requiring shell augmentation; implant-driven treatment planning)
2	Was the condition measured in a standard, reliable way for all participants?	Defect type and site (horizontal, vertical, mixed; maxilla/mandible) were assessed using clinical and radiographic criteria applied consistently across cases
3	Were valid methods used for identification of the condition for all participants?	Ridge deficiencies were diagnosed using clinical examination and standardized radiographs/CBCT prior to augmentation
4	Did the case series have consecutive inclusion of participants?	Studies were evaluated for consecutive or clearly defined recruitment (e.g., “all patients treated between year X and Y”). Lack of clarity was rated as “Unclear”
5	Did the case series have complete inclusion of participants?	The completeness of inclusion over the stated time period and reasons for any exclusions were assessed
6	Was there clear reporting of the demographics of the participants in the study?	Age, sex distribution, and defect location (maxilla/mandible; anterior/posterior) were extracted where available
7	Was there clear reporting of clinical information of the participants?	Baseline defect characteristics, augmentation indication, and any relevant systemic factors were recorded when reported
8	Were the outcomes or follow-up results of cases clearly reported?	Outcomes such as bone gain, resorption, implant survival, and complications were considered clearly reported when measurement methods and time points were specified
9	Was there clear reporting of the presenting site(s)/clinic(s) demographic information?	Single-center vs multicenter setting, country, and clinical context (private practice/university clinic) were documented where stated
10	Was the follow-up long enough for outcomes to occur?	Follow-up duration for augmentation stability and implant survival was evaluated relative to accepted time frames (e.g., ≥6 to 12 months after loading)
11	Was there clear reporting of the intervention(s) or treatment procedure(s)?	Details of plate type (allogeneic/autogenous), graft mixture, fixation, membranes, and healing protocols were required for a “Yes” rating
12	Were the statistical methods well described?	Descriptive and, where applicable, inferential statistics (e.g., means, SDs, p-values) had to be reported clearly for a “Yes” rating
